# Integrated disease surveillance and response in humanitarian context: South Sudan experience

**DOI:** 10.11604/pamj.supp.2022.42.1.33779

**Published:** 2022-06-17

**Authors:** John Rumunu, Joseph Francis Wamala, Sheila Baya Konga, Alice Lado Igale, Abraham Abenego Adut, Scopas Korsuk Lonyik, Robert Martin Lasu, Rose Dagama Kaya, Guyo Guracha, Peter Nsubuga, Fabian Ndenzako, Olushayo Oluseun Olu

**Affiliations:** 1 Directorate of Preventive Health Services, South Sudan Ministry of Health, Joint Doctoral Program in Global Health, Humanitarian Aid and Disaster Medicine, Universita Del Pemonte Orientale and Vrije University Brussel, Juba, South Sudan,; 2 World Health Organization Country office, Juba, South Sudan,; 3 Global Public Health Solutions, Juba, South Sudan

**Keywords:** Disease surveillance, priority diseases, outbreak investigation, emergency preparedness and response, data analysis, South Sudan

## Abstract

**Introduction:**

decades of instability continue to impact the implementation of the Integrated Disease Surveillance and Response (IDSR) strategy. The study reviewed the progress and outcomes of rolling out IDSR in South Sudan.

**Methods:**

this descriptive cross-sectional study used epidemiological data for 2019, 2020, and other program data to assess indicators for the five surveillance components including surveillance priorities, core and support functions, and surveillance system structure and quality.

**Results:**

South Sudan expanded the priority disease scope from 26 to 59 to align with national and regional epidemiological trends and the International Health Regulations (IHR) 2005. Completing the countrywide rollout of electronic Early Warning Alert and Response (EWARS) reporting has improved both the timeliness and completeness of weekly reporting to 78% and 90%, respectively, by week 39 of 2020 in comparison to a baseline of 54% on both timeliness and completeness of reporting in 2019. The National Public Health Laboratory confirmatory testing capacities have been expanded to include cholera, measles, HIV, tuberculosis (TB), influenza, Ebola, yellow fever, and Severe Acute Respiratory Syndrome 2 (SARS-COV-2). Rapid response teams have been established to respond to epidemics and pandemics.

**Conclusion:**

since 2006, South Sudan has registered progress towards using indicator and event-based surveillance and continues to strengthen IHR (2005) capacities. Following the adoption of third edition IDSR guidelines, the current emphasis entails maintaining earlier gains and strengthening community and event-based surveillance, formalizing cross-sectoral one-health engagement, optimal EWARS and District Health Information Systems (DHIS2) use, and strengthening cross-border surveillance. It is also critical that optimal government, and donors’ resources are dedicated to supporting health system strengthening and disease surveillance.

## Introduction

Public health security is essential to minimizing the impact of acute public health events and is critical for countries’ social and economic stability and transformation [1]. Health security is even more critical in fragile humanitarian settings given the constrained health and social services and the vulnerabilities to disease outbreaks and other public health emergencies, hence the high likelihood of excess morbidity and mortality. The International Health Regulations (IHR, 2005) mandate countries to establish and maintain effective surveillance and response systems to preserve national and international public health security [2]. The Integrated Disease Surveillance and Response (IDSR) is a strategy for strengthening national public health surveillance and response systems at all levels and provides a framework for attaining the IHR (2005) core capacity requirements in the African region [3,4]. Member states in the WHO African region adopted the strategy in 1998.

Numerous years of civil strife in South Sudan and inadequate public health and healthcare investments have contributed to the increased risk of outbreaks and insufficient capacities to detect and respond to public health threats. Within this context, in 1998, South Sudan experienced a relapsing fever outbreak that is estimated to have resulted in more than 400,000 cases, including 2,000 deaths (CFR 5 0.5%) [5]. The Early Warning Alert and Response Network (EWARN) was established in 1999 within Operation Lifeline Sudan (OLS) initiative to ensure timely detection and prompt response to similar public health threats. The IDSR strategy was eventually launched in 2006 to replace and expand EWARN [5]. However, systematic IDSR implementation started in 2009 after securing grants from USAID and ECHO through the World Health Organization (WHO) [6].

Since the adoption of IDSR by the WHO Africa (WHO-AFRO) Region in 1998, the strategy has been implemented successfully in stable countries. The strategy has been used to improve capacities to respond to cholera, meningitis, Ebola Virus Disease (EVD) and other outbreaks or public health events. In this present paper, we share the experiences and impact of using IDSR to strengthen national surveillance and response systems in a country experiencing a protracted grade 3 humanitarian crisis that has persisted since 2014 with a scale that warrants major support from WHO and other United Nations Agencies [7]. These experiences will inform the updating of current guidance on implementing the IDSR strategy in fragile settings and thus reduce the risk of excess morbidity and mortality.

## Methods

This was an observational, descriptive cross-sectional study that used the quantitative program and aggregate epidemiological data for all the epidemiological weeks in 2019 and 2020 to document the progress of implementing IDSR and building national surveillance and response capacities in South Sudan. Additionally, other IDSR program data from 2006, were compared with program benchmarks to document the implementation progress. The study used Ministry of Health (MoH) IDSR and Early Warning Alert and Response Network (EWARN) data collected routinely at the health facility, county, and state levels or from periodic program monitoring reports during the study period. Program reports, including monitoring, training, support supervision, and periodic assessment reports, were reviewed to obtain the information on the program performance indicators (Annex 1). The surveillance system components assessed include the availability of a list of priority diseases, conditions, and events for surveillance; the structure of the surveillance system; the core and support functions; and the quality of the surveillance system (Annex 1) [8]. We identified sub-components and relevant indicators for demonstrating progress on the capacity benchmarks established under the IDSR strategy (Annex 1) [8]. We used existing routine MoH aggregate data and coded case-based data with no personal identifiers. We collated the data on surveillance performance indicators into the study data abstraction tool.

**Annex 1:** integrated disease surveillance and response indicator matrix

No	Component	Element	Indicator	Indicator definition	Target	Data source/ method
1.1	PH priorities targeted for surveillance	Prioritizing PH events for surveillance	Objectives for disease surveillance	Exitance of objectives for national disease surveillance	Every 5-10 years	Surveillance guidelines, plan
1.2	PH priorities targeted for surveillance	Prioritizing PH events for surveillance	Disease prioritization	Evidence of prioritization of diseases for surveillance	Every 5-10 years	Surveillance guidelines, plan
1.3	PH priorities targeted for surveillance	Prioritizing PH events for surveillance	Updated priority diseases list	Number of years since the last update of the priority disease list	Every 5-10 years	Surveillance guidelines, plan
2.1	Structure	Surveillance legislation (laws and regulations)	Legislative support for the implementation of surveillance and response	Existence of legislation for surveillance and response	Existence of PH legislation	Document review (JEE report)
2.2	Structure	Surveillance legislation (laws and regulations)	Decree or orders for surveillance and response	Existence of a decree or order for surveillance and response	Existence of decree or order for PHSR	Document review (outbreak response)
2.3	Structure	Compliance with IHR (2005)	Presence of national IHR focal point	Designated IHR focal point	existence of national IHR FP	Document review (JEE report)
2.4	Structure	Compliance with IHR (2005)	Functioning IHR communication facilities	Evidence of functional email/telephone at IHR focal point for international notification and reporting	existence of communication facilities	Document review (JEE report)
2.5	Structure	Compliance with IHR (2005)	Timely notification to WHO of outbreaks of international importance	Proportion of outbreaks of international concern that were notified to WHO within 24 hours of detection	80-100%	Document review (JEE report)

The periodic indicator measures were compared to the program targets during the study period to document quantitative and qualitative changes that demonstrate program performance concerning set indicator-specific targets. Quantitative changes in program performance indicators entailed absolute measurements, proportions, or rates. These descriptive analyses were undertaken in Microsoft Excel. We used aggregate data from routine Ministry of Health epidemiological bulletins and program reports with no personal identifiers, and therefore, an ethical review was not warranted for this present study.

Ethics approval and consent to participate: administrative clearance for publication of this paper was provided by the Ministry of Health of South Sudan and WHO (WHO e-Pub no: ePub-IP-00331294-EC).

## Results

We present the performance on indicators aligned to the five surveillance system components starting with the surveillance priorities, then the core and support functions, and surveillance system structure and quality.

**Surveillance priorities:** South Sudan adopted the IDSR strategy in 2006, but systematic implementation aimed at establishing national surveillance structures aligned to the strategy started in 2009. These initiatives were further buttressed when South Sudan became a WHO Member State on 27 September 2012 and after the country committed to the IHR (2005) on April 16, 2013.

**Objectives for disease surveillance:** the national surveillance goals are aligned to the IHR (2005) and the IDSR strategy for the African region 2020-2030. Thus, the South Sudan Ministry of Health adapted the third edition IDSR guidelines and training materials during a 5-day WHO-facilitated workshop from October 21, 2019. The Government eventually adopted these guidelines on November 13, 2019 thus paving the way for their dissemination and use by counties, health facilities and communities. Based on these guidelines, the main objective of the national surveillance system is to improve the country´s capacities to detect, report, confirm, and effectively respond to priority diseases, conditions, and events. The updated guidelines are explicit on one-health surveillance for zoonotic diseases, the use of eHealth to enhance surveillance, cross-border surveillance strengthening, and surveillance in humanitarian contexts.

**Prioritization of diseases for surveillance:** as part of the revised 2019 national IDSR guidelines, the priority list was updated from the 26 diseases, conditions, and events prioritized in the 2012 guidelines to 59 diseases, conditions, and 19 events for indicator and event based surveillance (Annex 2 and Annex 3). The broad categories include diseases, conditions, or epidemic-prone events, diseases targeted for eradication or elimination; other major diseases of public health importance; and diseases or events of international concern (Annex 2 and Annex 3). The top cause of morbidity in South Sudan in 2019 was malaria that accounted for 75.9% of outpatient consultations and hence is top on the priority list (Table 1). The additional diseases prioritized include vaccine-preventable diseases like pertussis, chickenpox, and suspect rabies, as cases have been on the rise in under-vaccinated populations after emerging as major causes of morbidity and mortality in recent years (Table 2).

**Annex 2:** integrated disease surveillance and response list of priority diseases, conditions, and events

Number	Category	Disease, condition, or event
1	Epidemic prone diseases and/or reportable on a weekly basis	Malaria (suspected and confirmed)
2		Cholera 1
3		Acute watery diarrhoea
4		Bacterial meningitis
5		Diarrhoea with blood (Shigella)
6		Viral haemorrhagic fevers*1
7		Dengue
8		Typhoid fever
9		Yellow fever 1
10		Measles
11		Chickenpox
12		Diphtheria
13		Pertussis (whooping cough)
14		Influenza-like illness (ILI)
15		Severe Acute Respiratory Infection (SARI)
16		Plague 1
17		Relapsing fever
18		Nodding syndrome
19		Brucellosis
20		Zika
21		Coronavirus disease 2019 (COVID-19)
22	Epidemic prone diseases and/or reportable on a weekly basis	Acute Jaundice Syndrome
23		Animal bites (suspect rabies)
24		Snake bites
25		Visceral leishmaniasis (Kala azar)
26		Anthrax
27		Chikungunya
28		Maternal deaths
29		Perinatal deaths
30		Adverse Events Following Immunization
31		Presumptive Multi Drug Resistant TB (MDR-
32		Gunshot/shell injury
33		Other injury
34		Skin disease (scabies, etc.)
35		Malnutrition
36	Diseases targeted for eradication or elimination	Dracunculiasis (Guinea worm)
37		Leprosy
38		Neonatal tetanus
39		Poliomyelitis 1 (AFP)
40		Onchocerciasis
41		Lymphatic filariasis
42	Other major diseases of public health importance	Diarrhoea in children <5
43		Pneumonia <5
44		HIV new cases
45		Tuberculosis
46		Sexually Transmitted Infections (STIs)
47		Human African Trypanosomiasis (HAT)
48		Buruli ulcer
49		Schistosomiasis
50		Soil Transmitted Helminths (STH)
51		Trachoma
52		Acute viral hepatitis
53		Hypertension
54		Diabetes mellitus
55		Epilepsy
56	Diseases or events of international concern	Human influenza due to a new subtype
57		Severe Acute Respiratory Syndrome (SARS)
58		Smallpox
59		Any public health event of international or national concern (infectious, zoonotic, food borne, chemical, radio nuclear, or due to
Immediate notification and weekly reporting		
Epidemic prone diseases Targeted for elimination or eradication Events of unknown cause or potential PHEICs National/international requirement Monthly reporting		
All the other diseases, events of public health importance are reported on monthly		

**Annex 3:** integrated disease surveillance and response list of events under event-based surveillance.

Number	Category of event	Disease, condition, or event (for immediate reporting)
1	Human	Clustered human cases of disease or syndromes
2		Unusual disease patterns
3		Unexpected cluster deaths at community level
4		Events that constitute potential public health risk to humans
5		Unusual drug consumption patterns
6		Student absenteeism because of illness
7		Death due to suspect epidemic prone disease
8		Death of a mother or new-born
9		Any other public health event of national or international concern: (Infectious, zoonotic, foodborne, chemical, or radiological, unknown
10		Stock out on 50% of tracer medicines at HF
11	Animal and environmental	Bleeding in livestock or wildlife
12		Unusual animal deaths
13		Unusual deaths of birds - domestic or wild
14		Unusual deaths of fish
15		Abortion in small animals (goats, sheep etc.)
16		**Emergencies: (**floods, population displacement, fires, landslides, earthquakes, extensive crop failure or crop diseases/pests, mass casualty events)
17		Toxic dump on land or water body
18		Hand pump breakdown
19		Attacks on health worker, health facilities or other health resources

**Table 1:** top causes of morbidity in South Sudan in 2019

Disease or syndrome	Morbidity in week 52, 201		Cumulative morbidity for all weeks in 2019	
	Number of cases	Proportional morbidity [%]	Number of cases	Proportional morbidity [%]
Malaria	28,373	60.8%	2,728,314	75.9%
ARI	8,610	18.5%	335,190	9.3%
AWD	5,753	12.3%	440,650	12.3%
Bloody diarrhoea	879	1.9%	73,688	2.1%
AJS	4	0.0%	681	0.0%
Measles	160	0.3%	3,208	0.1%
Other	2,866	6.1%	12,403	0.3%
Total cases	46,645	100%	3,594,134	100%

**Table 2:** selected diseases that emerged from 2017 to 2019

#	Disease	Cases	Deaths	CFR	Attack rate per 10,000	Year	Location
1	Pertussis (probable)	10	0		0.91	2019	Leer
2	Chickenpox	38	0		2.49	2019	Awerial
3	Chickenpox	2701	0		91.13	2017	Wau
4	Suspect rabies	38	1	2.6%	6.18	2018, 2019	Nzara
5	Suspect rabies	679	0		84.85	2019	Agok (Abyei)

CFR - case fatality ratio

### Structure of Integrated Disease Surveillance and Response

*Legislation to facilitate surveillance:* the IDSR strategy has been incorporated into the National Health Policy (2016-2026) and the National Health Sector Development Plan (2017-2022) to facilitate the implementation of IDSR in South Sudan. However, the public health bill and the animal health bill have not been enacted as Acts of Parliament as of writing this report.

*Legislation to facilitate outbreaks and emergency response:* the overall mandate for regulation and provision of healthcare and health emergency response is constitutionally vested in the national and state governments. Consequently, the Minister of Health has sanctioned the formation of national and state-level outbreak task force committees to control outbreaks of cholera, measles, hepatitis E, and other public health events in recent years. In the same way, presidential orders were issued to establish the National COVID-19 task force to provide the overall policy, strategic, and oversight guidance and coordination of the national COVID-19 response.

### Compliance with International Health Regulations (2005)

*Designation of National International Health Regulations Focal Point:* in line with the IHR (2005) requirement, the office of the Director General International Health and Coordination in the National Ministry of Health is the designated IHR (2005) National Focal Point (NFP) for coordinating IHR (2005) core functions implementation in collaboration with other sectoral focal points.

*Functioning International Health Regulations communication facilities:* while the National IHR Focal Point has been designated, staffing and communication logistics remain inadequate for the office to meet functional and communication objectives. Official communication email and telephone facilities have not been designated for efficient communication with the other sectors and the WHO IHR focal point.

*International notification of outbreaks to World Health Organization:* in compliance with the IHR (2005), an outbreak of yellow fever in Sakure, Nzara County and measles outbreaks in 20 counties were notified to WHO as potential Public Health Emergencies of International Concern (PHEIC) in 2019.

### Surveillance strategy and coordination

**Figure 1:**
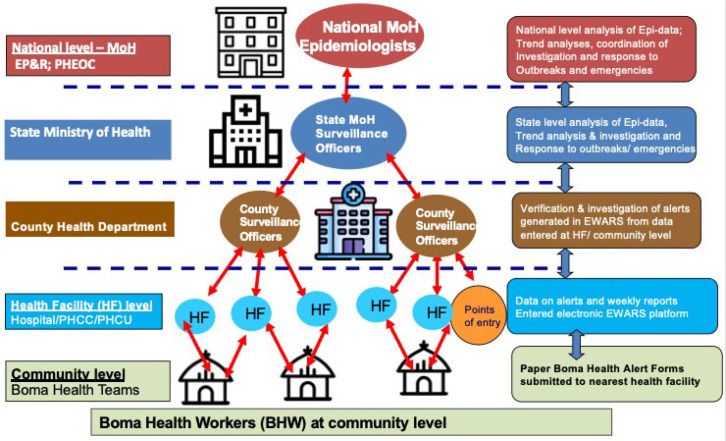
integrated disease surveillance and response functions by health system level in South Sudan

*National Integrated Disease Surveillance and Response and Early Warning Alert and Response Network Coordination:* the Emergency Preparedness and Response (EP&R) department under the Directorate of Preventive Health Services in the national MoH is the designated IDSR coordination unit at the national level. It is supported by state-level surveillance focal points in the respective state MoH (Figure 1). Within the states, county surveillance officers in respective county health departments (CHDs) support IDSR functions at the county, health facility and community level (Figure 1). All health facilities, including the partner supported EWARN clinics, report to and are supported by the respective county and state-level surveillance officers. The national EP&R department provides oversight, policy, quality assurance, and strategic guidance to implement IDSR in the country.

*The emergency preparedness and response committee and its roles in integrated disease surveillance and response coordination:* the EP&R department is also the secretariat of the national EP&R committee. The EP&R committee comprises Public Health Officers, Laboratory focal point, and partners from all the programs mandated to manage the priority diseases under surveillance, the National Public Health Laboratory (NPHL) and the health promotion department. The committee also provides strategic and operational guidance on strengthening IDSR core and support functions at all levels. The EP&R committee convenes weekly to review and support preparedness for anticipated outbreaks and public health emergencies; coordinates initial investigations and responses to new and ongoing suspect or confirmed outbreaks and other public health emergencies. In 2019, there were 14 (27%) documented weekly EP&R meetings with minutes on the record.

*Evidence of sharing resources:* to optimize IDSR functional capacities at all levels, integrated use of resources occurs through detection and reporting of acute flaccid paralysis (AFP), Guinea worm, other vaccine-preventable diseases (VPDs) to the respective vertical programs for case-based investigations. The integrated use of IDSR rapid response teams (RRTs) and surveillance focal points to investigate and respond to AFP, Guinea worm disease (GWD), and other VPDs. The use of IDSR/EWARS reporting resources to support reporting needs under the DHIS2 has also been initiated. Further, influenza sentinel surveillance resources under IDSR have been used to support Ebola virus disease, yellow fever, and COVID-19 investigations and laboratory testing.

### Networking and partnerships

*Intersectoral collaboration, networking, and partnership:* effective control of outbreaks like cholera, Rift Valley Fever, Ebola virus disease, and COVID-19 requires the competencies of a designated intersectoral committee for systematic risk assessment, contingency planning, and effective response. However, South Sudan lacks a formalized multisectoral platform to take on this role. Moreover, recent intersectoral engagements are timebound and restricted to the active phase of outbreaks and emergencies. During the cholera outbreaks of 2014-2017, the Ministry of health and partners worked alongside the Ministry of water and irrigation and Juba City Council to implement water quality surveillance and improve access to safe water sources. In the same way, the Ministry of Livestock and Fisheries (MLF) has worked with the Ministry of Health and partners under the task force constituted to respond to the Rift Valley Fever (RVF) outbreak of 2017.

*Existence of functional laboratory networks:* South Sudan has one National Public Health Laboratory (NPHL) that was founded in 1974 but was not inaugurated until 2014 and has capacities to test for cholera, measles, human immunodeficiency virus (HIV), tuberculosis (TB), influenza, Ebola, Marburg, yellow fever, severe acute respiratory syndrome coronavirus 2 (SARS-COV-2), and routine water quality testing (Table 3). The laboratory reagents and supplies required to support the NPHL to conduct the tests availed through partners like WHO with funding support from USAID, ECHO, and Country-Based Pooled Funds (CBPF); UNDP with funding support from the Global Fund; and CDC with funding from The President´s Emergency Plan For AIDS Relief (PEPFAR). There are currently only two molecular laboratories at the sub-national level (Nimule and Wau). Hence, most of the samples are shipped using United Nations (UN) and Non-Governmental Organization (NGO) humanitarian flights and, to a lesser extent, by commercial carries to Juba, where the NPHL is situated. Public health testing is either done at NPHL or designated WHO international collaborating laboratories. Since measles was the most frequent outbreak reported in 2019 from 20 counties, we used it as a proxy for assessing the capacity of NPHL to process samples efficiently. The median turnaround time for measles samples from the field to the NPHL in 2019 varied from one day to 123 days. While the median turnaround time for testing measles samples in 2019 was 5 days and ranged from 1 day to 129 days. The capacities for antimicrobial resistance, monitoring of food safety, and routine external quality assurance are critical and need to be established as required under IDSR and IHR (2005).

**Table 3:** laboratory sample testing for selected priority diseases in 2019

No.	Disease (test)	Number of samples			Comments
		Tested	Positive	Negative	
1	Vibrio cholerae (culture)	134	0	134	
2	Measles (ELISA)	671	313	358	Of the 358 samples that tested negative for measles, 155 tested positive for Rubella IgM
3	Influenza (PCR)	309	31	228	
4	Ebola (PCR)	30	0	37	
5	Yellow fever	41	3		
6	Hepatitis E virus (PCR)	71	57	14	

ELISA - enzyme-linked immunosorbent Assay; PCR - polymerase chain reaction

*Cross-border collaboration:* the EAC cross-border surveillance framework was promulgated in 2011, with South Sudan joining in 2017 and participating in annual cross-border meetings since 2018. Cross-border surveillance zones and committees were formed to foster information sharing, regular meetings, joint training, simulations, investigations, and response. During the cross-border surveillance meeting held from 24 to 26 April 2018 in Nimule, South Sudan, the ninth zonal cross-border committee was formed as a tripartite zone covering Kenya, South Sudan, and Kenya. This cross border committee includes surveillance, laboratory, public health, and veterinary officers from the 19 border districts or counties (one district in Kenya; eight counties in South Sudan; and 11 districts in Uganda). The committee members share disease surveillance information regularly, and a social media platform was created to complement the other official communication channels. The tripartite cross-border surveillance committee meets annually and has conducted one joint cross-border outbreak response simulation exercise on the Ebola virus in 2019. The committee has also conducted two cross-border outbreak investigations on Rift Valley Fever and yellow fever in 2019 and 2020, respectively. The later investigation resulted in the confirmation of a yellow fever outbreak in Kajo-keji county in March 2020. Synchronized yellow fever vaccination campaigns were implemented on either side of the international border by October 2020. Alongside Uganda, the Democratic Republic of Congo (DRC), and Uganda, South Sudan has participated in the Goma cross-border surveillance initiative. The initiative strengthened cross-border surveillance to mitigate the risk of cross-border spread during the 2018/ 2019 Ebola virus disease (EVD) outbreak in North Kivu and Ituri, DRC. South Sudan is also participating in intercountry meetings to strengthen regional health security. In May 2019, WHO-AFRO convened an intercountry meeting in Kigali, Rwanda, to strengthen regional health security for operational readiness and surveillance in response to the EVD outbreak in DRC. The meeting involved the four high-risk countries (i.e., Rwanda, Uganda, Burundi, and South Sudan) bordering DR Congo.

### Performance on core functions

*Case detection and registration:* South Sudan is implementing both indicator and event-based surveillance to detect and register new cases of priority diseases or other public health events. As part of strengthening indicator-based surveillance, case definition charts and booklets have been printed and disseminated for use at health facilities. In 2019, 3,540 case definition booklets and charts were printed and distributed to the 1,260 functional health facilities. This number of case definition job-aids translates into at least three case definition job-aids distributed per functional health facility. As part of community-based surveillance, the Boma health workers (BHW) and community health workers (CHW) use simplified and translated community case definitions for case detection and registration at the community level. These simplified community case definitions are included in the case definition booklets. Indicator-based surveillance is complemented by event-based surveillance, which entails informal alerts of potentially serious public health events from the community, health facility, county, state, or national level. The log of rumors and suspect outbreaks is used for recording new alerts or suspected outbreaks. In 2019, at least one log of rumors and suspect outbreaks was distributed per functional health facility. A national toll-free center hotline exists in the call center and is used to report community alerts. Additionally, the electronic Early Warning Alert and Response (EWARS) reporting platform includes an event based platform that facilitates alert recording, verification, and risk assessment by health facility, county, and state surveillance officers and watch officers in the Public Health Emergency Operations Center (PHEOC).

**Figure 2:**
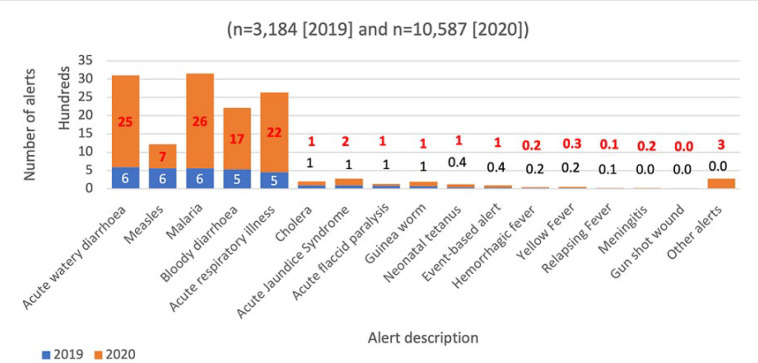
number of alerts by disease, South Sudan, 2019

In 2019, 3,184 alerts were detected and reported through the national event-based disease surveillance system using EWARS. The alerts reported in 2019 are four times higher when compared to the alerts (771) reported in 2018 (Figure 2). The significant increase in alert detection is attributed to the countrywide rollout of EWARS to the health facilities that has enhanced the detection and transmission of alerts countrywide and thus increased the capacity to detect outbreaks. Out of the 3,184 alerts reported in 2019, a total of 2,302 (72%) were verified by surveillance officers at the health facility, county, and state levels. Out of the 3,184 outbreak alerts reported, 18% were attributed to acute watery diarrhoea; 17% to suspected measles, malaria, and acute bloody diarrhoea; and 14% to acute respiratory infections.

*Case confirmation and response to outbreaks and other public health emergencies:* during 2019, at least 104 suspect outbreaks were reported and investigated, with 35 (33.65%) confirmed. Of the 35 confirmed outbreaks; 4 (11.4%) were due to Hepatitis E virus; 20 (57.1%) were due to measles; 9 (25.7%) were due to rubella;1 (2.9%) due to yellow fever; and 1 (2.9%) due to mass casualty (burns) (Table 4). Since the onset of the crisis in South Sudan in 2013, cholera cases have been reported every year since 2014. From 2014-2017 at least 28,676 cases, including 644 deaths (case fatality rate [CFR] 2.2%), were reported in South Sudan, but no cholera cases were confirmed in 2018, 2019, and 2020. In 2019, 105 stool samples were collected from suspect cholera cases, but they all tested negative for Vibrio cholerae after microbiological culturing and biochemical testing.

**Table 4:** distribution of confirmed outbreaks in South Sudan in 2019

No	Event	Location	Cases	Deaths	CFR%	Attack rate (cases per 10,000)
1	Yellow fever	Sakure	3	0	0.0%	0.5
2	Rubella	Aweil Center	35	0	0.0%	3.1
3	Rubella	Bor South	4	0	0.0%	0.2
4	Rubella	Gogrial West	5	1	20.0%	0.1
5	Rubella	Yirol East	3	0	0.0%	0.3
6	Rubella	Malakal PoC	23	0	0.0%	9.4
7	Rubella	Yirol West	19	0	0.0%	1.1
8	Rubella	Bentiu PoC	51	0	0.0%	4.9
9	Rubella	Wau PoC AA	11	0	0.0%	8.5
10	Rubella	Malakal PoC	178	0	0.0%	72.9
11	Measles	Abyei	316	0	0.0%	39.5
12	Measles	Juba	58	3	5.2%	1.0
13	Measles	Pibor	2056	9	0.4%	91.5
14	Measles	Gogrial West	156	0	0.0%	4.0
15	Measles	Mayom	23	0	0.0%	1.2
16	Measles	Aweil South	33	0	0.0%	2.9
17	Measles	Melut	9	0	0.0%	0.3
18	Measles	Gogrial East	11	0	0.0%	0.7
19	Measles	Aweil Center	23	0	0.0%	2.0
20	Measles	Malakal PoC	30	0	0.0%	12.3
21	Measles	Rumbek East	82	3	3.7%	3.7
22	Measles	Bor PoC	3	0	0.0%	15.5
23	Measles	Bentiu PoC	51	0	0.0%	4.9
24	Measles	Aweil East	19	0	0.0%	0.4
25	Measles	Wau PoC AA	436	5	1.1%	336.4
26	Measles	Renk	7	0	0.0%	0.3
27	Measles	Tonj North	20	2	10.0%	0.8
28	Measles	Tonj South	47		0.0%	3.6
29	Measles	Yambio	16	1	6.3%	0.7
30	Measles	Jur River	61	1	1.6%	3.2
31	Hepatitis E virus	Rubkona	36	1	2.8%	3.4
32	Hepatitis E virus	Lankien	1	0	0.0%	0.1
33	Hepatitis E virus	Aweil Center	1	0	0.0%	0.1
34	Hepatitis E virus	Lankien	12	0	0.0%	0.7
35	Burns (mass casualty)	Aweil North	130	36	27.7%	4.5

*Reporting, data analysis and feedback:* all priority diseases and events should be reported to the next high level either immediately, weekly, or monthly to facilitate appropriate public health action. Since the adoption of IDSR in South Sudan in 2006, routine IDSR reporting relied on a combination of paper-based, Microsoft Excel or Access datasets transmitted by email or radio calls to the next level. These approaches were fragmented, incomplete, and were associated with reporting errors and delays. Hence, in 2017, WHO, through the Global EWARS project, initiated support to the Ministry of Health and partners to streamline reporting. The support entailed modular training and technical support to facilitate the Early Warning Alert and Response System (EWARS) deployment. The EWARS is a web-based desktop and mobile application that can be rapidly configured and deployed to support early warning, alert management, and outbreak response. The EWARS mobile reporting has been deployed to support reporting in at least 1,260 functional health facilities in 10 States and three administrative areas. As part of the EWARS rollout from January 2019 to March 2020, WHO, in collaboration with the local governments and partners, has trained over 1,500 frontline health workers and distributed 21 “EWARS in a box” kits to facilitate real-time submission routine IDSR and disease outbreak reports. Each EWARS in a box kit contains 60 mobile phones with SIM cards, 25 solar power banks, and 60 EWARS quick start guides.

**Figure 3:**
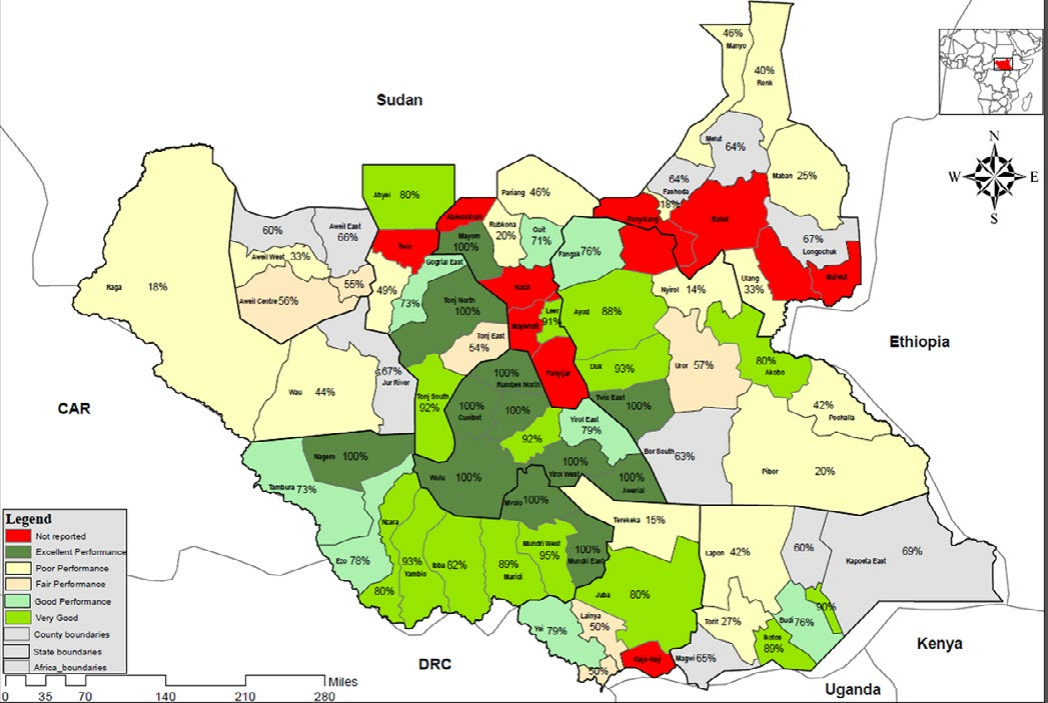
completeness in weekly reporting by county in week 39, 2019

**Figure 4:**
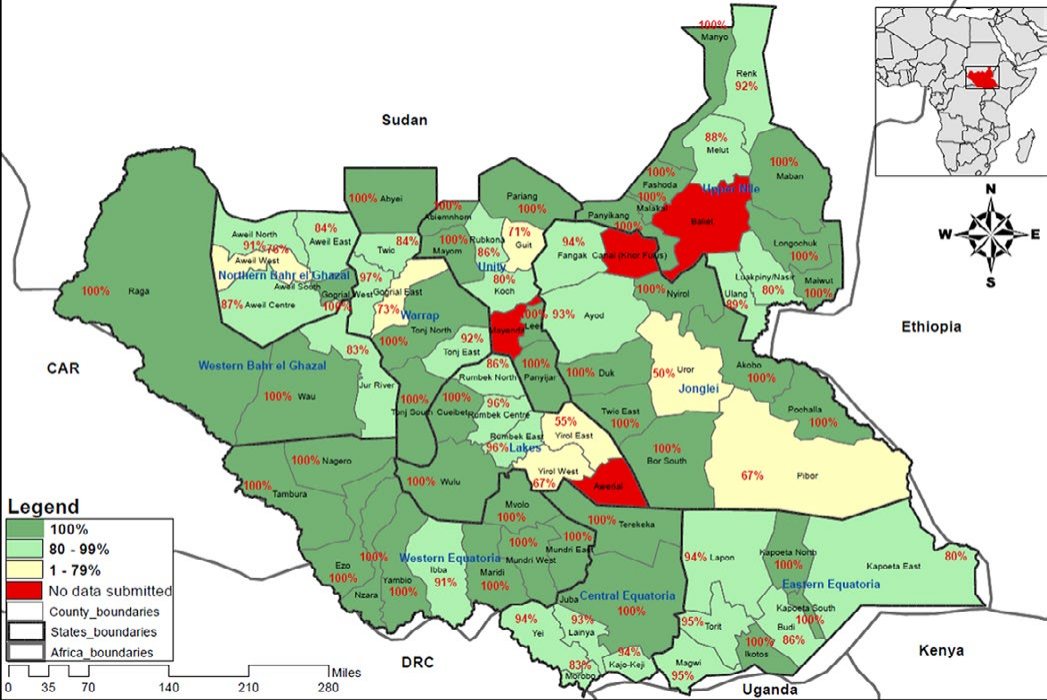
completeness in weekly reporting by county in week 39, 2020

The completion of electronic EWARS reporting rollout has positively impacted the timeliness and completeness of weekly IDSR reporting from the health facilities. The average timeliness for submitting weekly IDSR reports from the health facilities by the end of week 39, 2020 was 78% (947 of 1,221 health facilities) compared to 54% (807 of 1,491 health facilities) by week 39, 2019, representing an increase of 24%. In the same way, the average completeness rate of reporting from the functioning health facilities across the country by week 39, 2020 was 90% (1,094 out of 1,221 functional health facilities) as compared to 54% (807 out of 1,491 functional health facilities) in week 39, 2019 representing an increase of 36%. Overall, the completeness of weekly reporting from the health facilities by county improved in 2020 when compared to 2019 and 2020 (Figure 3, Figure 4). Early warning alert and response system is programmed to generate and disseminate automated weekly national and state-level epidemiological bulletins that include indicators on the weekly reporting performance, alert management, and trends on the number of cases and deaths for the priority diseases and public health events. The Ministry of Health IDSR bulletin is produced and disseminated weekly. It contains updates on the performance of the surveillance system and updates on detection, investigation, and response to new and ongoing outbreaks and public health events. However, analysis and utilization of IDSR data is weak or nonexistent and needs to be strengthened at the county and health facility levels. All the 52 (100%) weekly bulletins for 2019 were produced and published.

*Epidemic preparedness:* epidemic preparedness and contingency plans are developed and updated within national and state-level efforts to improve public health security. The updated preparedness and contingency plans include cholera, epidemic meningitis, yellow fever, Rift Valley Fever, and floods. Emergency funds are incorporated into the overall MoH budget to facilitate the implementation of the plans for providing primary health care as defined in the basic package for nutrition and health (BPNH). Epidemic preparedness and response is also incorporated into humanitarian response in prioritized populations and supported using the Country-Based Pooled Funds (CBPF) and through the ongoing support to IDSR implementation by ECHO and USAID.

*Response and control:* with support from the WHO, the MoH has established a national multi-hazards rapid response team and state rapid response teams in each of the 10 states with regular multi-hazard training and simulation exercises. The operations of the rapid response teams are guided by standard operating procedures that define the team composition, roles of each team member, and the procedure for activation, mobilization, and deployment in response to suspect and confirmed outbreaks. Once an outbreak is confirmed, the Minister of Health designates a task force committee to coordinate the overall response in liaison with other sectors and stakeholders.

**Performance on support functions:** in November 2019, the South Sudan MoH adapted and adopted the third edition of IDSR technical guidelines. The guidelines are expanded to include guidance on event-based surveillance, cross-border surveillance, the use of information technology to enhance surveillance functions, guidance on surveillance in the humanitarian context, and the one-health approach. The initial training of trainers on the third edition IDSR technical guidelines have been completed. The cascade county county-level training plan has been finalized though the implementation was delayed and modified in the context of COVID-19. A dedicated national IDSR plan of action is updated annually and incorporates all the activities required to implement the support functions and strengthen the core functions at all levels. However, the plan of action is primarily donor-supported, thus raising sustainability issues in the medium to long term.

## Discussion

The present study assessed performance for five core surveillance components to document the impact and experiences of deploying IDSR in a humanitarian context. Through the implementation of the IDSR strategy, South Sudan has registered significant progress towards strengthening the national disease surveillance system as demonstrated from the performance on key surveillance indicators for each of the surveillance components assessed in the present study.

**Objectives of disease surveillance:** in line with the IHR (2005) and the regional strategy for IDSR 2020-2030, the South Sudan Government adopted the IDSR to expand and strengthen the national surveillance and response system. Over the years, the Government adapted the requisite guidelines, expanded the priority disease list, and provided technical oversight to establish surveillance focal points, train and equip them, and monitor the implementation of IDSR functions at all levels [4]. However, the gap between country IDSR capacities and national targets is still significant, with only 26% of countries in the WHO African region having optimal IDSR implementation at the peripheral level [9]. Achieving national IDSR and IHR (2005) targets requires sustained Government stewardship, the appropriation of ample resources from the national budget to support public health security and health system strengthening, as well as social, economic, and political stability [10,11]. Since the initiation of systematic implementation of IDSR in 2009, the program has remained heavily dependent on donor resources. Therefore, it is critical that budget allocation to health security and health systems strengthening is propped up if donor investments over the recent years are to be sustained.

**IDSR structure:** the South Sudan Ministry of Health has established coordination structures and technical focal points to optimize IDSR functions at the community, health facility, county, state, and national level. The cross-sectoral engagement has been exploited in response to recent cholera outbreaks (2014-2017), Rift Valley Fever (2017), and COVID-19. However, these efforts are timebound and confined to the active outbreak phase. Furthermore, these efforts need to be formalized and should be extended to span the entire epidemic and emergency management cycle. Hence, as part of the NAPHS, there are plans to formalize cross-sectoral linkages with relevant line Ministries [12]. Further, the enabling laws on public health and animal health have been prioritized for enactment to facilitate public health response [12].

**Existence of laboratory networks:** laboratory capacities are critical to the IDSR function of detecting and confirming public health hazards [4]. South Sudan has a nascent public health laboratory system that was only inaugurated in 2014 with initial capacities to test cholera, bacterial meningitis, measles, and HIV [13]. Over the years, these capacities have been expanded to include TB, influenza and COVID-19, yellow fever, Ebola, and Marburg. Since these capacities are only available at the national level, these capacities must be extended to the sub-national level [12]. This will allow higher volumes of samples to be tested per capita, reduce the turnaround for specimen testing, and facilitate prompt response to outbreaks and public health events.

**Cross border collaboration:** the regional outbreaks of EVD in West Africa in 2014 spread to at least five countries in West Africa. Moreover, exported cases were reported in Europe and the United States in 2014 and 2015. Cross border EVD spread was again reported during the 2018 DRC Ebola outbreak in Ituri and North Kivu that spread to Uganda. These incidents highlight the need to enhance cross-border surveillance and response [14]. In this regard, therefore, South Sudan is currently participating in several regional cross-border initiatives. The EAC cross-border initiative is well established and involves South Sudan, Uganda, Kenya, Tanzania, Rwanda, and Burundi [15]. A total of nine cross-border surveillance zones are in place to facilitate regular emergency preparedness planning, information sharing, simulations in the EAC. South Sudan has continued to participate in the regular annual cross-border meetings since 2018 [15]. These efforts align well with the regional IDSR strategy for enhancing health security [4].

**Performance on core and support functions:** in line with the third edition IDSR technical guidance, South Sudan is expanding community and health facility-based surveillance using the indicator and event-based surveillance [4,9]. Boma Health Workers report alerts to the nearby health facility or use the dedicated national toll-free hotline for verification at the community level. To reinforce healthcare level capacities, the health workers training and distribution of guidelines have been undertaken. Still, health workers trained so far fall short of the optimal 2-3 trained health workers per functional health facility [4]. Gaps in training and access to requisite guidelines have been identified as impediments to IDSR implementation in Northeastern Nigeria [16]. Reporting of public health events is critical for prompt initiation of public health response. The rolling out of mobile EWARS reporting has improved the health facility IDSR reporting performance and feedback to all levels. The suboptimal IDSR reporting performance in some counties is related to the impact of impact of recurrent cycles of insecurity, hazards like floods, inadequate access to mobile telecommunications network and internet on the functionality of the health information system. The institutionalization of multidisciplinary rapid response teams has optimized investigation and response capacities at all levels. The other functions critical to optimizing the current gains entail regular emergency preparedness and response meetings, support supervision missions to state, county, and health facility level and provision of communication resources like phones, power banks and computers. South Sudan, like other countries, continues to report gaps in these key surveillance and outbreak response functions as seen from the successive of waves of outbreaks like cholera [17-19]. A notable achievement from these efforts entails cholera response from 2014-2017, with no new cholera outbreaks confirmed in South Sudan in 2018, 2019, and 2020. This success is attributed to the mapping of cholera hotspots where surveillance was enhanced in the IDSR context, case management, access to safe water and sanitation and hygiene were enhanced alongside the deployment of safe cholera vaccines [17,20].

The limitations of this study entail incomplete program and epidemiological data and the cross-sectional nature of the study that is not ideal for demonstrating the program’s impact. Therefore, we used multiple sources of data to triangulate information and reduce data gaps. Also, we used standardized program performance indicators to document progress on core and support IDSR functions.

## Conclusion

Since the adoption of IDSR in 2006, South Sudan has registered progress towards strengthening the national disease surveillance system. In compliance with the IHR (2005) and IDSR, the priority disease scope has been expanded, with indicator and event-based surveillance used to detect and report alerts and suspect cases. In addition, the countrywide rollout of electronic EWARS reporting has improved the reporting performance. Further, laboratory capacities continue to be strengthened following the inauguration of the National Public Health Laboratory, which now possesses capabilities for confirming priority bacterial and viral pathogens supported by a countrywide network for specimen referral. To facilitate investigation and response to suspect and confirmed outbreaks, national and state rapid response teams have over the years been trained, drilled, and deployed to support investigation and response to outbreaks of cholera, Rift Valley Fever, yellow fever, viral hemorrhagic fevers, and COVID-19. Epidemic preparedness is effectively supported through the national EP&R team. In contrast, the response to confirmed outbreaks is supported by task force committees of experts from varied disciplines and sectors using the incident management system (IMS) principles. The rollout of the third edition IDSR guidelines will further bolster this progress. The rollout will facilitate formal cross-sectoral one-health engagement, enable optimal use of EWARS and DHIS2, and allow effective cross border engagement. In collaboration with the health cluster partners, the MoH will continue using the IDSR to address fragile, vulnerable, and conflict-affected populations’ surveillance and response needs. To ensure that these investments in IDSR are optimized and sustained, budget support towards strengthening the health system and surveillance system must be optimized. To address the challenge of high healthcare workers turnover, the working conditions of health workers in public health facilities must be enhanced. The NAPHS, which embodies IHR (2005) and IDSR priorities, should be implemented to enhance national and international health security. Finally, as the country works towards emerging from decades of conflict and economic downturn, donor support to the IDSR program must be sustained to reduce morbidity and mortality from priority health diseases, conditions, or events and enhance national and international health public health security.

### What is known about this topic

- The integrated disease surveillance and response (IDSR) strategy provides a framework that countries in the WHO African region are using to attain core capacity requirements for the international health regulations (IHR (2005)). The member states in the African region have demonstrated that IDSR is a cost-effective strategy for effective preparedness, detection, investigation, response and control of epidemics and pandemics in the African region.

### What this study adds

- Whereas the IDSR strategy has largely been used in stable settings, in this paper, we have demonstrated how the IDSR functions can be used to support Early Warning Alert and Response (EWARN) in humanitarian settings where the performance of surveillance systems is sub optimal. The sustained investment of resources towards IDSR implementation has improved the program reporting performance, thus allowing detection of major outbreaks, and averting morbidity and mortality;
- Program funding from domestic resources is critical for sustainability in the long-term.
